# Characteristics of a Novel Target Antigen against Myeloma Cells for Immunotherapy

**DOI:** 10.3390/vaccines8040579

**Published:** 2020-10-02

**Authors:** Maiko Matsushita, Saku Saito, Shinya Yokoe, Daiju Ichikawa, Yutaka Hattori

**Affiliations:** Division of Clinical Physiology and Therapeutics, Faculty of Pharmacy, Keio University, Tokyo 105-8512, Japan; saku.vn19@gmail.com (S.S.); shinyayokoe@gmail.com (S.Y.); ichikawa-di@pha.keio.ac.jp (D.I.); hattori-yt@pha.keio.ac.jp (Y.H.)

**Keywords:** myeloma, antigen, demethylating agents

## Abstract

Despite the availability of therapeutic treatments, multiple myeloma is an incurable haematological disorder. In this study, we aimed to clarify the role of CXorf48 as a therapeutic target in multiple myeloma. Based on a previously identified HLA-A*24:02-restiricted epitope from this novel cancer/testis antigen, we characterized the activities of cytotoxic T lymphocytes (CTLs) specific to this antigen against myeloma cells and evaluated the effects of demethylating agents in increasing antigen expression and enhancing the cytotoxic activity of CTLs. CXorf48 expression was examined by reverse transcription polymerase chain reaction (RT-PCR) using nine myeloma cell lines. Cell lines with low CXorf48 expression were treated by demethylating agents (DMAs), 5-azacytidine (5-aza), and 5-aza-2’-deoxycytidine (DAC) to evaluate gene expression using quantitative RT-PCR. Furthermore, CXorf48-specific CTLs were induced from peripheral blood mononuclear cells of HLA-A*24:02-positive healthy donors to evaluate antigen recognition using ELISpot and ^51^Cr cytotoxicity assays. CXorf48 was widely expressed in myeloma cells, and gene expression was significantly increased by DMAs. Furthermore, CXorf48-specific CTLs recognized DMA-treated myeloma cells. These findings suggest that CXorf48 is a useful target for immunotherapy, such as vaccination, in combination with demethylating agents for the treatment of patients with myeloma.

## 1. Introduction

Multiple myeloma is a plasma cell neoplasm accompanied by various symptoms, including anaemia, renal dysfunction, lytic bone lesion, and hypercalcemia [1,2]. Despite the development of various therapeutic drugs, such as immune modulatory drugs (IMiDs) and proteasome inhibitors, and treatment strategies, including high-dose chemotherapy and stem cell transplantation, which have prolonged the median survival of multiple myeloma patients, it remains difficult to cure myeloma patients [3]. In recent years, antibodies, antibody-drug conjugate (ADC), bispecific T-cell engager (BiTE), or chimeric antigen receptor (CAR)-T cells have also been found to be effective treatment options, suggesting that myeloma cells are highly sensitive to immunotherapies [4,5].

Recent technological developments, including multiparameter flow cytometry or next-generation sequencing techniques, have enabled the detection of minimal residual disease (MRD) in myeloma patients [6]. The meta-analysis of several clinical studies has shown that MRD negativity is associated with a better clinical prognosis [7]. Therefore, eradicating MRD is essential to cure myeloma patients. However, most myeloma patients are elderly, and prolonged therapy, with its severe adverse effects, is often not suitable.

Vaccination with tumor-specific antigens has been reported to reduce MRD in cancer patients, without inducing severe toxicities [8]. For myeloma patients, vaccinations using myeloma-specific peptide antigens or dendritic cells pulsed with antigens or fused with myeloma cells have been investigated, and have been found to enhance anti-myeloma immunity in preclinical settings [9,10]. However, a high therapeutic efficacy is needed to obtain tangible results in clinical settings.

In this study, we aimed to clarify the role of CXorf48 as a novel therapeutic target in multiple myeloma. In a previous study, we found this antigen to be expressed in chronic myelogenous leukaemia cells, and not expressed in normal tissues, except the testes. We also previously identified a human leukocyte antigen (HLA)-A*24:02-restiricted epitope from this novel cancer/testis antigen (CTA) [11]. In the present study, the expression of this antigen in myeloma cells was detected, and cytotoxic T lymphocyte (CTL) activity against myeloma cells was evaluated. In addition, the effects of demethylating agents, including 5-azacytidine (5-aza) or 5-aza-2’-deoxycytidine (DAC), were examined for their role in increasing antigen expression and enhancing the cytotoxic activity of myeloma cell lines with low levels of CXorf48 expression. Our findings suggest that CXorf48 is a useful target for immunotherapy in combination with demethylating agents for the treatment of myeloma patients.

## 2. Materials and Methods

### 2.1. Cell Lines

KMM1, KMS11, KMS20, KMS21, KMS26, KMS27, KMS28, and KMS34 were kindly provided by Dr. Ohtsuki of Kawasaki Medical School (Kurashiki, Japan). MUM24 was established in our laboratory from a thalidomide-resistant patient [12]. CIR-A24 cells were kindly gifted by Dr. Yutaka Kawakami of Keio University, School of Medicine (Tokyo, Japan). All of these cell lines were established from Japanese myeloma patients. The leukaemia cell line K562 was purchased from the Health Science Research Resources Bank (National Institute of Biomedical Innovation, Osaka, Japan). U266 was obtained from American Type Culture Collection. Cells were maintained at 37 °C in a humidified atmosphere of 5% CO_2_ in RPMI-1640 medium (Sigma-Aldrich, Saint Louis, MO, USA) and 10% foetal bovine serum (FBS) (Invitrogen™, Life Technologies, Grand Island, NY, USA).

### 2.2. Reagents

Phytohemagglutinin (Sigma-Aldrich) was used for generation of phytohemagglutinin (PHA) blasts in CTL induction. 5-azacytidine and 5-aza-2’-deoxycytidine (Sigma-Aldrich) were dissolved in dimethyl sulfoxide (DMSO) and stored at −20 °C. Peptide derived from CXorf48 (DYGMIDESI) and peptide derived from Human Immunodeficiency Virus (HIV) (RYLRDQQLL) were synthesized at purification of 98% by high-performance liquid chromatography (HPLC) (Sigma-Aldrich, Tokyo, Japan). Purified peptides were dissolved in DMSO and stored in aliquots at −80 °C.

### 2.3. Detection of CXorf48 Gene Expression by RT–PCR

Expression of CXorf48 gene was detected by the standard reverse transcription polymerase chain reaction (RT–PCR) and quantitative PCR, as previously described [11]. Total RNA was extracted from myeloma cell lines, K562, and peripheral blood mononuclear cells (PBMNC) from healthy donor (HD) using Isogen (Nippon Gene Co. Ltd., Tokyo, Japan). Complementary DNA was made from 1 μg of the total RNA by ReverTra Ace qPCR RT Master Mix with gDNA Remover (Toyobo, Osaka, Japan) according to manufacturer’s instructions. Then, PCR with CXorf48 primers (forward, 5′-gttgtgcctcgccatctttatg-3′ and reverse, 5′-tgcactggggtgatagaaatcg-3′) or *GAPDH* primers (forward, 5′-tgaacgggaagctcactgg-3′ and reverse, 5′-tccaccaccctgttgctgta-3′) was conducted using Taq polymerase (Takara Bio, Shiga, Japan). Quantitative PCR was conducted using SsoFast probes supermix (Bio-Rad, Hercules, CA, USA) using CXorf48 primers and probe (TaqMan Gene Expression Assays; Applied Biosystems, CA, USA) or *GAPDH* primers (forward, 5′-gacctgacctgccgtctagaaa-3′and reverse, 5′-cctgcttcaccaccttcttga-3′) and probe (5′-(6-FAM)-acctgccaaatatgatgac-(BHQ-2)-3′). Complementary DNA from the K562 cell line was used to make standard curves. The relative gene expression level was calculated as follows: CXorf48 expression level (sample)/*GAPDH* expression level (sample). Written informed consent was obtained for the use of blood samples from heathy donors. This study was approved by the Ethics Committee of Keio University Faculty of Pharmacy (190613-10).

### 2.4. Detection of Cancer/Testis Antigen (CTA) Gene Expression by RT-PCR

The expression of the CTA genes *NY-ESO-1*, *MAGE-A1*, *MAGE-A2, MEGE-A3*, *MAGE-C1*, *LAGE-1*, and *PRAME* was detected by RT-PCR using gene-specific primers (*NY-ESO-1*: forward, 5’-CCCCACCGCTTCCCGTG-3’ and reverse, 5’-CTGGCCACTCGTGCTGGGA-3’; *MAGE-A1*: forward, 5’-CGGCCGAAGGAACCTGACCCAG-3’ and reverse, 5’-GCTGGAACCCTCACTGGGTTGCC-3’; *MAGE-A2*: forward, 5’-AAGTAGGACCCGAGGCACTG-3’ and reverse, 5’-GAAGAGGAAGAAGCGGTCTG-3’; *MEGE-A3*: forward, 5’-TGGAGGACCAGAGGCCCCC-3’ and reverse, 5’-GGACGATTATCAGGAGGCCTGCt-3’; *MAGE-C1*: forward, 5’-GACGAGGATCGTCTCAGGTCAGC-3’ and reverse, 5’-ACATCCTCACCCTCAGGAGGG-3’; *LAGE-1*: forward, 5’-CTGCGCAGGATGGAAGGTGCCCC-3’ and reverse, 5’-GCGCCTCTGCCCTGAGGGAGC-3’; *PRAME*: forward, 5’-CTGTACTCATTTCCAGAGCCAGA-3’ and reverse, 5’-TATTGAGAGGGTTTCCAAGGGGTT-3’).

### 2.5. Detection of CXorf48 Protein Expression by Immunocytochemical Staining

Cells were attached to glass slides using a Cytospin 4 cytocentrifuge (Thermo Fisher Scientific, Waltham, MA, USA), fixed with 2% paraformaldehyde for 20 min, permeabilized with Triton X-100, and blocked with 1% bovine serum albumin in PBS for 30 min. The samples were then stained with mouse anti-CXorf48 antibody (Sigma-Aldrich), washed with PBS (Sigma), and stained with Alexa Fluor488 goat-anti-mouse IgG (Molecular Probes, Thermo Fisher Scientific, Eugene, OR, USA) and washed with PBS (Sigma).

### 2.6. Treatment with Demethylating Agent

KMS11, KMS34, and peripheral blood mononuclear cells (PBMNC) from healthy donors (HDs) were cultured in RPMI-1640 medium (Sigma-Aldrich) supplemented with 10% FBS (Invitrogen) in 6-well culture plates. Then, 200 nM of 5-azacytidine or 5-aza-2’-deoxycytidine (Sigma-Aldrich) was added to these cells every 24 h for 72 h.

### 2.7. Generation of CXorf48-Specific CTLs from Human PBMNC

CXorf48-specific *CTLs* were generated by in vitro stimulation with peptide-pulsed autologous dendritic cells and PHA blasts, as previously described [13]. Briefly, PBMNC were isolated from the whole peripheral blood obtained from HLA-A*24:02-positive healthy donors by Ficoll density gradient centrifugation. Monocyte-derived dendritic cells (DC) were generated from CD14^+^ cells selected by a MACS separation system (Miltenyi Biotec, Bergisch Gladbach, Germany). The purified CD14^+^ cells were cultured in AIM-V medium supplemented with 10% AB human serum, 100 ng/mL of IL-4 (R&D Systems), and 100 ng/mL of Granulocyte Macrophage colony-stimulating Factor (GM-CSF) (R&D Systems), for 5 days. Then, 20 ng/mL of TNF-α (R&D Systems) was added to generate DC. PHA blasts were generated from CD14^−^ cells by culturing in AIM-V medium supplemented with 10% human serum, 100 units of IL-2, and 1 μg/mL of PHA, for 2 days. The DCs or PHA blasts were pulsed with 50 μg/L of peptide at room temperature for 3 h before irradiation (mediXtec, Chiba, Japan). On day 0, the CD14^−^ cells were stimulated with irradiated peptide-pulsed DC in AIM-V medium with 10% human serum supplemented with 10 ng/mL of IL-7 (R&D Systems). Then, 50 ng/mL of IL-2 was added every 2 to 3 days. Those cells were stimulated weekly with irradiated peptide-pulsed autologous PHA blasts. Then, CD8^+^ cells were purified using immunomagnetic beads specific for CD8 (Miltenyi Biotec). Written informed consent was obtained for use of blood samples from heathy donors. This study was approved by the Ethics Committee of Keio University Faculty of Pharmacy (190613-10).

### 2.8. Detection of IFN-γ Secretion from CTLs by Enzyme-Linked Immunospot (ELISpot) Assay

The IFN-γ secreted from CTLs against target cells was detected using ELISpot assay. Briefly, effector cells were co-cultured with target cells in 96 well plates coated with anti-IFN-γ antibody (1-D1K; Mabtech Inc., Cincinnati, OH, USA). After incubation for 20 h, a biotinylated antibody specific for IFN γ (7-B6-1; Mabtech) was added and incubated for 2 h at room temperature, followed by the addition of streptavidin-alkaline phosphatase (Mabtech) for 1 h. Nitroblue tetrazolium and 5-bromo-4-chloro-3-indolyl phosphate (BioRad) were added to develop spots. Color development was stopped by washing with distilled water. The resulting spots were counted using a CTL-ImmunoSpot1 analyzer (Cellular Technology Ltd., Shaker Heights, OH).

### 2.9. Detection of Cytotoxicity of CTLs by Cytotoxicity Assay

The cytotoxic activity of the CTLs was measured using a standard ^51^Cr release assay. The target cells were labelled with 50 μCi of ^51^Cr (PerkinElmer Inc., Waltham, MA, USA) for 60 min at 37 °C. The labelled target cells were co-cultured with effector cells. After 4 h of incubation, the resulting supernatants were transferred to LumaPlates (PerkinElmer) and allowed to air dry overnight. The plates were sealed and counted using a Plate CHAMELON V (Hidex, Turku, Finland). The percentage of lysis specific to ^51^Cr release was calculated as follows: [(experimental ^51^Cr release−spontaneous ^51^Cr release)/(maximum ^51^Cr release−spontaneous 51Cr release)] × 100.

### 2.10. Dextramer Staining

CTLs induced by stimulation with the CXorf48-derived peptides were washed with PBS (Sigma), stained with antibodies against CD3 (33-2A3; Immunostep, Salamanca, Spain), CD8 (BW135/80; Miltenyi Biotec), and dextramer specific for HIV-derived peptide or CXorf48-derived peptide (Immudex, Copenhagen, Denmark). The resulting cells were analyzed using flow cytometry (LSR II; BD Biosciences, Franklin Lakes, NJ, USA).

### 2.11. Statistical Analysis

The results are presented as the mean ± standard error of the mean (s.e.m.). Groups were compared using Student’s *t*-test. Differences were considered significant at *p* < 0.05.

## 3. Results

### 3.1. CXorf48 Is Expressed in Myeloma Cells

**Figure 1:**
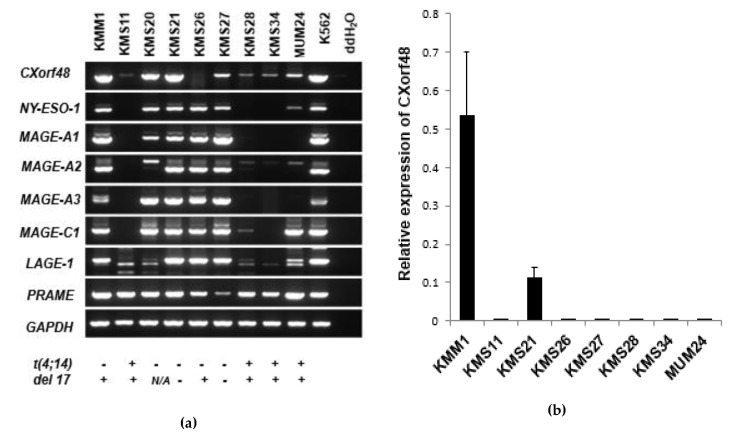
CXorf48 gene expression in multiple myeloma (MM) cell lines. (**a**) Expression of CXorf48 gene and other cancer-testis antigen (CTA) genes in nine myeloma cell lines were detected using conventional reverse transcription polymerase chain reaction (RT-PCR). K562 was used as a positive control. Chromosomal abnormalities were indicated below. N/A; not analyzed. (**b**) Expression of CXorf48 genes in myeloma cell lines was evaluated by quantitative PCR. Relative expression was calculated by dividing expression level of CXorf48 by that of *GAPDH*. K562 was used as a positive control to make standard curves. Data are mean with standard deviations.

We first evaluated the levels of CXorf48 gene expression in myeloma cell lines, including KMM1, KMS11, KMS20, KMS21, KMS26, KMS27, KMS28, KMS34, and MUM24, using RT-PCR (Figure 1a). Eight out of the nine cell lines were found to express this gene. Among these cell lines, KMM1, KMS26, KMS28, KMS34, and MUM24 harbored high-risk chromosomal abnormalities, such as *t* (4;14) or del17. We also detected the expression of other CTA genes, namely, *NY-ESO-1*, *MAGE-A1*, *MAGE-A2*, *MAGE-C1*, *LAGE-1*, and *PRAME*. *MAGE-A1* and *MAGE-A3* were found to be commonly expressed in five myeloma cell lines (KMM1, KMS20, KMS21, KMS26, and KMS27), while *NY-ESO-1* was detected in six cell lines (KMM1, KMS20, KMS21, KMS26, KMS27, and MUM24), and *MAGE-A2* and *MAGE-C1* were detected in seven cell lines (KMM1, KMS21, KMS26, KMS27, KMS20, and MUM24). *LAGE-1* and *PRAME* were expressed in all of the cell lines.

Quantitative RT-PCR revealed that KMM1 and KMS21 expressed high levels of CXorf48 (Figure 1b). By contrast, CXorf48 gene expression was not detected in KMS11 by quantitative PCR, although a small band was found to express CXorf48, detected using standard PCR, suggesting that KMS11 expresses extremely low levels of this gene.

**Figure 2:**
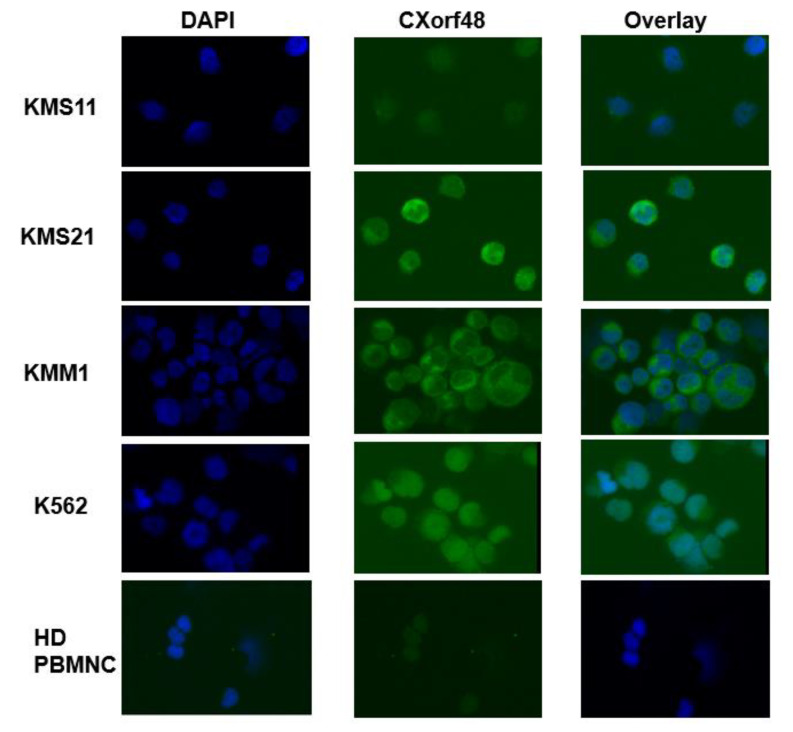
CXorf48 protein expression in MM cell lines. Expression of protein was detected using immunocytochemistry in KMS11, KMS21, KMM1, K562 (positive control), and peripheral blood mononuclear cells were detected in a healthy donor (HD PBMNC; negative control). These cells were stained with 4′,6-diamidino-2-phenylindole (DAPI) and mouse anti-CXorf48 antibody followed by staining with Alexa488-labeled goat anti-mouse IgG.

We then conducted an immunocytochemistry assay to confirm protein expression. The cytoplasm of KMS21 and KMM1 was strongly stained with anti-CXorf48 antibody (Figure 2). We detected low levels of CXorf48 protein expression in KMS11, consistent with the RT-qPCR results. Our previous study showed that the CXorf48 gene is not expressed in blood mononuclear cells or bone marrow mononuclear cells [11]. Immunocytochemistry assay also showed that human blood mononuclear cells were not stained with anti-CXorf48 antibody (Figure 2). Therefore, CXorf48 can be a myeloma-specific antigen.

### 3.2. CXorf48-Specific CTLs Recognized Myeloma Cells with High Expression of CXorf48

**Figure 3:**
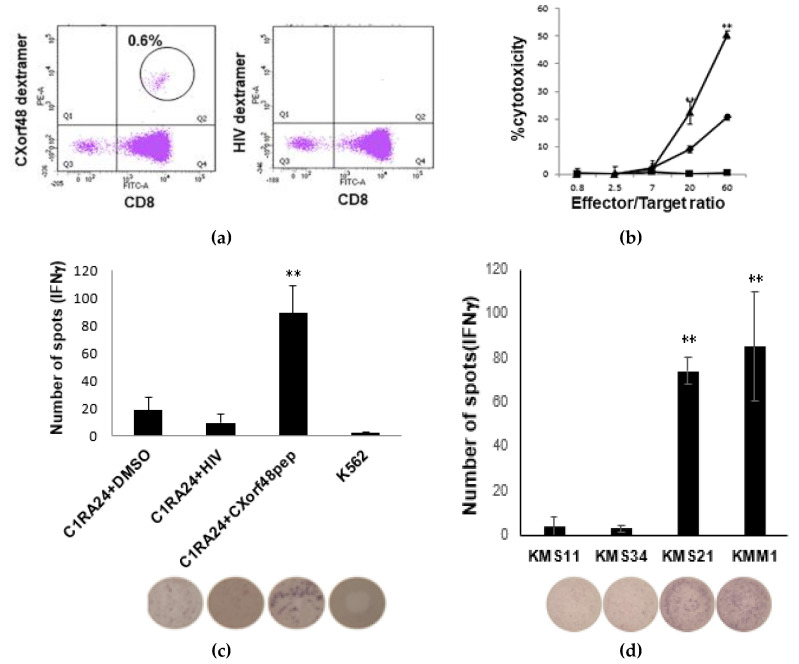
Activity of CXorf48-specific cytotoxic T lymphocytes (CTLs) against myeloma cells. (**a**) CXorf48-specific CTLs induced from peripheral blood mononuclear cells (PBMNC) from healthy donors using CXorf48^49–57^ peptide were stained with dextramer specific to CXorf48^49–57^ peptide (left) or dextramer specific to HIV-derived peptide (right), and analyzed by flow cytometer. (**b**) Cytotoxicity of CXorf48-specific CTLs against myeloma cell lines, KMS11 (HLA-A*24:02-positive, CXorf48-negative: ■), KMS21 (HLA-A*24:02-positive, CXorf48-positive: ▲), and K562 (HLA-A*24:02-negative, CXorf48-positive: ◆) was assessed by ^51^Cr release assay. (**c**,**d**) IFN-γ secretion by CTLs responding to CIR-A24 cells pulsed with CXorf48^49–57^ peptide or HIV-derived peptide, K562, and myeloma cell lines, KMS 11, KMS34, KMS21, and KMM1, was evaluated by ELISpot assay. Data are mean with standard deviations. ** *p* < 0.01 (Student’s *t*-test).

To assess the immunogenic property of CXorf48, we evaluated whether CTLs against CXorf48 were able to recognize myeloma cells. CXorf48-specific CTLs were induced from the PBMNC of HLA-A*24:02-positive healthy donors using the CXorf48^49–57^ peptide. After three stimulations with peptide-pulsed dendritic cells or PHA blasts, CD8^+^ cells were sorted and stained with dextramer containing a complex of the HLA-A*24:02 and CXorf48^49–57^ peptides. We found that 0.6% of CD3^+^ cells were positive for the antigen-specific dextramer (Figure 3a). Then, we assessed the cytotoxicity of the CTLs against myeloma cell lines. The CTLs recognized KMS21, which is HLA-A*24:02-positive with high levels of CXorf48 expression. On the other hand, the CTLs showed no cytotoxicity against KMS11, which is HLA-A*24:02-positive but has little CXorf48 expression. The CTL also did not lyse K562 cells, which lack HLA-A*24:02, with high levels of CXorf48 expression (Figure 3b). ELISpot assay confirmed the antigen-specific IFN-γ secretion from the CTLs. The CTLs secreted significantly high level of IFN-γ when co-cultured with CIR-A24 cells pulsed with CXorf48^49–57^ peptide, compared to CIR-A24 cells pulsed with irrelevant peptide (Figure 3c). We then detected IFN-γ secretion from CTLs against myeloma cells with or without CXorf48 expression (Figure 3d). The number of cells secreting IFN-γ was significantly higher when CTLs were cultured with KMS21 and KMM1, which have high levels of CXorf48 expression, compared to KMS11 or KMS34, which have low CXorf48 expression.

### 3.3. Up-regulation of CXorf48 Expression by Demethylating Agents

**Figure 4:**
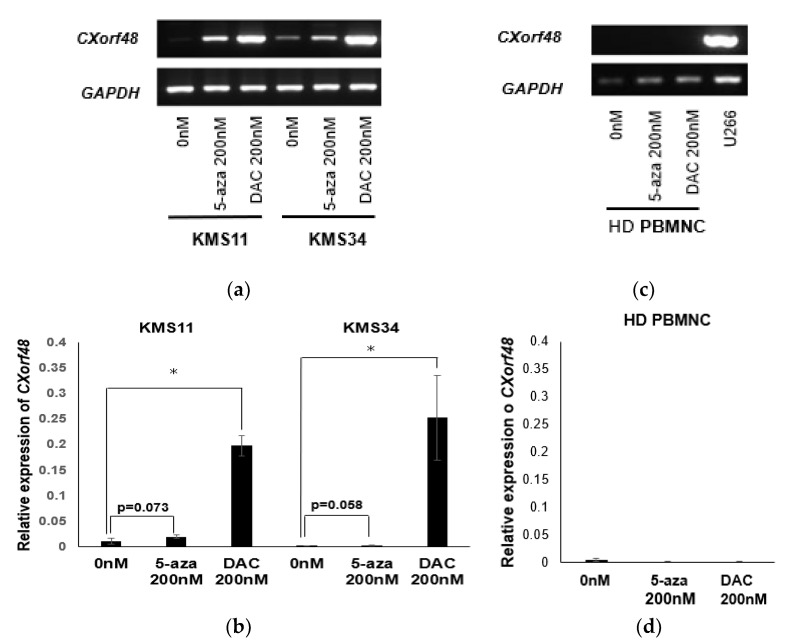
CXorf48 gene expression is up-regulated by treatment of demethylating agents. KMS11, KMS34, and peripheral blood mononuclear cells from healthy donor (HD PBMNC) were incubated with 200 nM of 5-azacytidine or 5-aza-2’-deoxycytidine for 72 h. RNA was extracted, and RT-PCR (**a**,**c**) or quantitative PCR (**b**,**d**) was performed. cDNA from U266 was used as a positive control for CXorf48 gene expression. Quantitative PCR was conducted in triplicate. Data are mean with standard deviations. * *p* < 0.05 (Student’s *t*-test).

A threshold amount of antigen is needed for recognition by cytotoxic T cells (CTLs). Therefore, increasing antigen expression in myeloma cells with low or no antigen expression may enhance the recognition of CTLs against a wide variety of myeloma cells. It has been previously reported that the gene expression of many CTAs is controlled by the methylation of their promoter regions [14,15]. Therefore, we examined whether CXorf48 expression is also up-regulated by demethylating agents in myeloma cells. As shown in Figure 4a, CXorf48 gene expression in KMS11 or KMS34 increased after treatment with 5-azacitidine (5-aza) or 5-aza-2’-deoxycytidine (DAC). Notably, treatment with DAC significantly up-regulated CXorf48 gene expression (*p* < 0.05) (Figure 4a,b), and 5-aza tended to increase CXorf48 gene expression (KMS11; *p* = 0.078, KMS34; *p* = 0.058). In contrast, gene expression was not detected in PBMCs from healthy donors, regardless of treatment with demethylating agents (Figure 4c,d).

### 3.4. CXorf48-Specific CTL Recognized DMA-Treated Myeloma Cells with Low CXorf48 Expression

**Figure 5:**
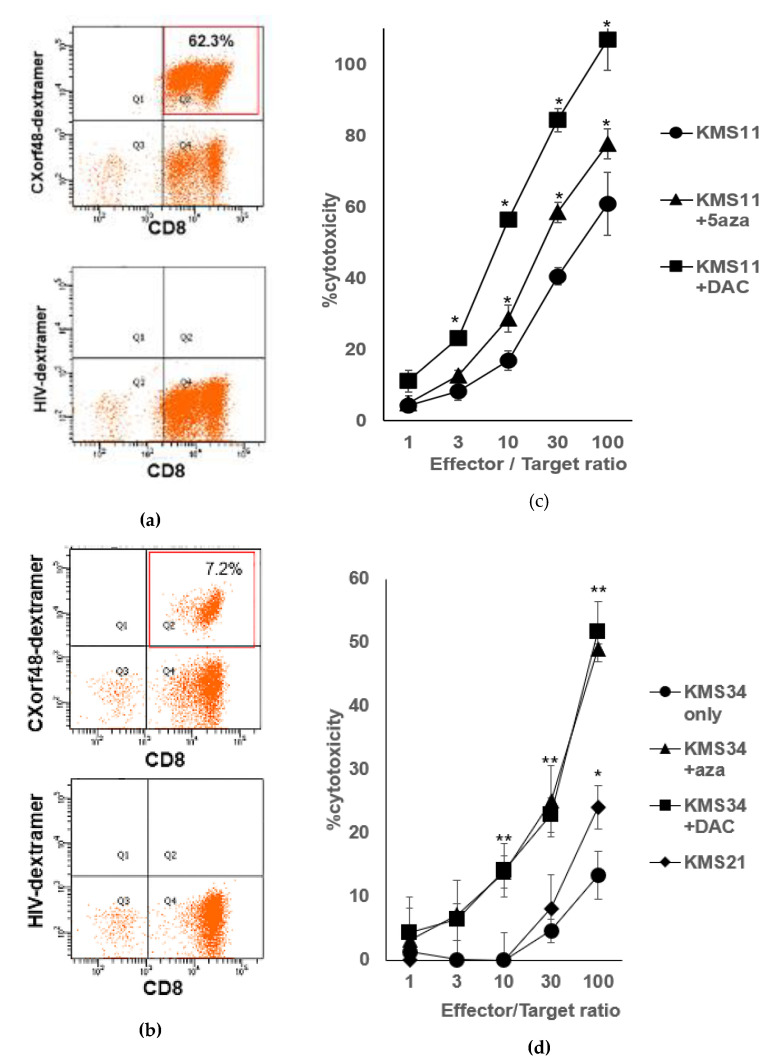
Activities of CXorf48-specific CTLs against demethylating agents-treated myeloma cells. (**a**,**b**) CXorf48-specific CTLs induced from PBMNC of healthy donors using CXorf48^49–57^ peptide were stained with dextramer specific to CXorf48^49–57^ peptide (upper) or dextramer specific to HIV-derived peptide (lower), and analyzed by flow cytometer. (**c**,**d**) Cytotoxicity of CXorf48-specific CTLs against myeloma cell lines, KMS11 or KMS34 (HLA-A*24:02-positive, CXorf48-negative) treated with DMSO, 5-aza, or DAC was assessed by ^51^Cr release assay. KMS21 (HLA-A*24:02-positive, CXorf48-positive) was used as a control. * *p* < 0.05, ** *p* < 0.01 (Student’s *t*-test, against KMS11 only or KMS34 only).

After finding that CXorf48 expression was up-regulated by demethylating agents in myeloma cells, we evaluated the activity of CXorf48-specific CTLs against CXorf48-low myeloma cells treated with demethylating agents. We induced antigen-specific CTLs from the PBMNC of healthy donors using the CXorf48^49–57^ peptide. As a result, 62.3% of the CD3^+^ cells were positive for dextramer specific to the CXorf48^49–57^ peptide (Figure 5a). These cells strongly lysed KMS11 treated with 5-aza or DAC, compared to non-treated KMS11 (Figure 5c). We also evaluated cytotoxicity of antigen-specific CTLs, which contained 7.2% of dextramer-positive cells, against KMS34 cultured with or without DMAs (Figure 5b,d) and observed that KMS34 treated with 5-aza or DAC was also more significantly damaged by CTLs than non-treated KMS34. These data suggested that the induction of CXorf48 in KMS11 or KMS34 allowed antigen-specific CTLs to recognize these originally CXorf48-negative myeloma cells.

## 4. Discussion

Although the development of immune checkpoint inhibitors and advances in next-generation sequencing technology have revealed the existence of neoantigens derived from somatic mutations in cancer cells, several types of cancers, including leukaemia, have only a small number of neoantigens, due to rare somatic mutations [16,17]. Multiple myeloma is known to be resistant to immune checkpoint inhibitors (ICIs) [18], suggesting that there are few neoantigens in this haematological malignancy. Therefore, shared cancer antigens highly expressed in myeloma cells are promising targets in immunotherapies for this disease, including vaccination using cancer-specific peptides [19,20]. However, vaccination using single antigen is not enough to elicit clinical benefits in cancer patients in some cases, and the use of multiple antigens is known to strengthen the effects of vaccination during the treatment of cancer or infectious diseases [21]. Therefore, clarification of different antigens to those previously reported is needed. In this study, we evaluated the role of CXorf48 as a novel immunological target in multiple myeloma.

CXorf48 is a CTA that is expressed in chronic myelogenous leukaemia cells and not in normal blood cells [11]. This antigen is also detected in some of solid cancers, including head and neck carcinoma [22]. However, its expression in myeloma cells has not yet been reported. Therefore, we evaluated the expression of CXorf48 in nine myeloma cells and found that most cells expressed this antigen. However, the expression levels varied among cell lines. While the KMM1, KMS20, KMS21, and KMS27 cell lines were found to highly express this gene, KMS11, KMS28, KMS34, and MUM24 showed low levels of expression. All the cells expressing low levels of CXorf48 (CXorf48-low cells) were found to have chromosomal abnormalities, such as *t* (4;14) and del17. However, the relationship between these abnormalities and the CXorf48 gene, which is located on the X chromosome, is still unknown. Translocation (4;14) is observed in 20% of myeloma patients, and causes the activation of the multiple myeloma SET domain-containing protein (MMSET), which in turn increases the methylation of lysine 36 in Histone H3, leading to the translation of other genes [23,24]. If *t* (4;14) is related to levels of CXorf48 expression, there is a possibility that this translocation promotes the expression of the suppressor gene of Cxorf48, although little is known about control of *Cxorf48* gene expression.

Immunocytochemical staining confirmed that CXorf48 protein is highly expressed in cytoplasm of KMM1 or KMS21. CTAs located inside the cells are processed by the proteasome, modified by the endoplasmic reticulum and Golgi apparatus, moved to cell surface, and finally presented by the HLA molecule. We previously identified an epitope, CXorf48^49–57^, which binds to HLA- A*24;02, the most popular HLA class I haplotype in the Japanese population [11]. *PRAME*, another CTA located in the 22nd chromosome [25], was also strongly expressed in all myeloma cell lines, which is consistent with reports that gene expression levels are lower in X chromosomal genes than in autosomal genes [26]. However, an epitope presented by HLA-A*24;02 has not been reported in *PRAME*, although the HLA-A*02;01-restricted epitope has been investigated and previously used in clinical trials [27]. This suggests that CXorf48 is an important immunological target in Japanese patients.

In order to clarify the immunogenicity of CXorf48 against myeloma cells, antigen-specific CTLs were induced from healthy donors, confirmed by antigen-specific dextramer staining. These CTLs were able to recognize myeloma cells, such as KMS21 and KMM1, with high levels of CXorf48 expression, but not those with low levels of antigen expression, suggesting that this antigen could be a therapeutic target, at least in myeloma patients expressing high levels of CXorf48.

To overcome this limitation, we tried to enhance antigen expression using DMAs, 5-aza, and DAC, since DMAs are known to increase the expression of several CTAs by lowering the methylation of their promotor region [14,15]. Both 5-aza and DAC were found to enhance CXorf48 expression in a myeloma-specific manner. Moreover, we evaluated the effect of DMAs on the recognition of myeloma cells by CTLs. Anti-CXorf48 CTLs showed a stronger cytotoxic activity against KMS11 or KMS34 treated with DMAs, which may be caused by up-regulated antigen expression in myeloma cells.

These results suggest that vaccination in combination with DMAs may be useful as effective immunotherapy for wide variety of myeloma patients. 5-aza and DAC have been approved for the treatment of myelodysplastic syndrome without severe side effects [28]. However, few reports exist on their use in myeloma patients [29]. In particular, DAC showed a more potent response compared to 5-aza in inducing both antigen expression and CTL recognition. DAC is known to have stronger effect of demethylation compared to 5-aza and does not affect the functions of T cells at low concentrations [15]. Recently, Zhou et al. reported that DAC could deplete myeloid-derived suppressor cells in vivo [30], suggesting that DAC may not only enhance CTL recognition of myeloma cells but also overcome immunosuppressive microenvironments. Furthermore, Wajnberg et al. reported that DAC treatment suppresses myeloma cell growth by suppressing the MYC oncogene [31]. DAC was found to increase the expression of TAZ, a transcriptional co-activator in Hippo-signalling pathway, via demethylating its promotor region, leading to decreased MYC expression. These data indicate that immunotherapy against CXorf48 with DAC could contribute to strong tumor regression in myeloma patients.

## 5. Conclusions

The findings of the present study suggest that CXorf48 has potential as a novel target antigen in the immunotherapy of multiple myeloma. Thus, a vaccination using CXorf48-derived epitope peptide or CXorf48 protein in combination with DMAs represents an attractive strategy for the treatment of patients with myeloma.
